# Characterization of SiGe thin films using a laboratory X-ray instrument

**DOI:** 10.1107/S0021889813010492

**Published:** 2013-06-07

**Authors:** Tatjana Ulyanenkova, Maksym Myronov, Andrei Benediktovitch, Alexander Mikhalychev, John Halpin, Alex Ulyanenkov

**Affiliations:** a Rigaku Europe SE, Am Hardtwald 11, Ettlingen, Germany; b Department of Physics, The University of Warwick, Coventry, UK; c Department of Theoretical Physics, Belarusian State University, Nezavisimosti Avenue 4, Minsk, Belarus

**Keywords:** thin films, high-resolution reciprocal space mapping, partly relaxed epitaxial films, misfit dislocation

## Abstract

The technique of reciprocal space mapping using X-rays is a recognized tool for the nondestructive characterization of epitaxial films. X-ray scattering from epitaxial Si_0.4_Ge_0.6_ films on Si(100) substrates using a laboratory X-ray source was investigated. It is shown that a laboratory source with a rotating anode makes it possible to investigate the material parameters of the super-thin 2–6 nm layers. For another set of partially relaxed layers, 50–200 nm thick, it is shown that from a high-resolution reciprocal space map, conditioned from diffuse scattering on dislocations, it is possible to determine quantitatively from the shape of a diffraction peak (possessing no thickness fringes) additional parameters such as misfit dislocation density and layer thickness as well as concentration and relaxation.

## Abstract 2

This article reports the characterization of thin SiGe/Si(100) epilayers using reciprocal space maps measured by a laboratory X-ray instrument and a high-resolution X-ray diffraction study of partially relaxed SiGe/Si thin films.

## 1. Introduction

Si substrates are widely used in the semiconductor industry owing to their low cost, good thermal conductivity, widespread availability and mature processing technology. In the past two decades, the growth of heterostructures on Si(100) substrates with different lattice constants from Si have been investigated. A relaxed SiGe buffer layer grown on the Si substrate can be used for further growth of lattice matched III–V heterostructures. The principle of the techniques is to use another layer between, for example, GaAs and Si or Ge and Si, which has an intermediate lattice parameter and coefficient of thermal expansion. For many practical reasons it is advantageous to have an SiGe buffer layer grown on Si(100), which is called a ‘virtual substrate’. Recently this technology has found effective applications as a platform for opto-, micro- and nanoelectronics. Fully strained pseudomorphic SiGe epilayers can also be grown directly on an Si(001) substrate and can be used as a high carrier mobility channel in MOSFET (metal-oxide semiconductor field-effect transistor) devices.

By control of the growth conditions one may grow epitaxically a smooth fully strained pseudomorphic SiGe layer on an Si substrate until the thickness of the layer reaches a critical thickness, *h*_c_, which depends upon both the germanium composition *x* and the growth temperature (Matthews & Blakeslee, 1974 ▶; Bolkhovityanov & Sokolov, 2012 ▶). If this critical thickness is exceeded, it becomes energetically favourable for the strain in the epilayer to be relieved through the formation of 60° *a*/2 misfit dislocations at the SiGe/Si interface.

A variety of techniques are used to characterize thin films, such as transmission electron microscopy (TEM), auger electron spectroscopy, high-resolution X-ray diffractometry (HRXRD) and X-ray reflectometry (XRR). The relative merits of different methods for thickness and concentration evaluation in SiGe layers have been considered by Zaumseil *et al.* (2004 ▶). X-ray characterization techniques are favourable because of their nondestructive nature. Reciprocal space mapping is often used to investigate the structural properties of epitaxial thin films (layer tilt, lattice relaxation, composition and quality of structures) (Zaumseil *et al.*, 2011 ▶), and the transition from the fully strained to the relaxed state can also be investigated with the help of reciprocal space maps (RSMs) (Benediktovitch *et al.*, 2011 ▶; Sasaki *et al.*, 2009 ▶). The XRR technique provides information about thickness, density and surface roughness. Accurate characterization of super-thin layers by X-rays using laboratory-based diffractometers is challenging owing to the low scattering intensity from a thin layer, so traditionally a synchrotron source is required.

In this work, we will use a laboratory X-ray source to characterize super-thin films. The paper has two main focuses: to demonstrate the capability of a laboratory X-ray source to investigate parameters of super-thin layers and to analyse quantitatively, by means of HRXRD, the microstructure of partially relaxed Si_0.4_Ge_0.6_ epilayers on Si(100) substrates. Super-thin Si_0.4_Ge_0.6_ epilayers on Si(100) substrates with thicknesses of 2–6 nm fully strained were used to demonstrate that the signal level from a laboratory instrument equipped with a 9 kW rotating anode Cu source is sufficiently powerful to obtain parameters of interest. Four partially relaxed samples of the same composition with thicknesses of 29, 50, 100 and 200 nm were investigated to demonstrate the capabilities of HRXRD in the presence of defects. The diffraction patterns from such structures (50–200 nm thickness) are formed mainly by diffuse scattering from misfit dislocations. The sample with 29 nm thickness is an example of an intermediate case, demonstrating the presence of diffuse and coherent scattering at the same time. A fast method to determine the relaxation and Ge content was suggested by Zaumseil (1994 ▶) on the basis of ω–2θ scans using Bragg–Brentano geometry. Based on the theory developed by Kaganer *et al.* (1997 ▶) it is possible to find quantitatively from the shape of a high-resolution RSM peak possessing no thickness fringes additional parameters such as misfit dislocation density and layer thickness.

## 2. Sample growth and measurement

Strained Si_0.4_Ge_0.6_ structures were grown on 200 mm-diameter Si(100) substrates in an industrial ASM Epsilon 2000 RP-CVD system. A thin constant-composition Si_0.4_Ge_0.6_ layer was grown at 723 K. During the growth, the wafer rotation speed remained constant and the chamber pressure was held below 100 Torr (1 Torr = 133.32 Pa).

HRXRD was used to determine the SiGe epilayers’ structural information. The SiGe epilayer thickness in each sample was also controlled by XRR and cross-sectional TEM.

The measurements were made using a 9 kW SmartLab Rigaku diffractometer with a rotating anode. A high-resolution setup with the combination of a two-crystal Ge monochromator in the 400 setting, a two-crystal Ge analyser in the same setting and a scintillation counter detector was used to achieve sufficient resolution for the measurement of a set of super-thin samples (thickness in the range 2–6 nm). The second set of samples was measured with a combination of a two-crystal Ge monochromator in the 400 setting and a D/teX Ultra detector (a one-dimensional high-speed position-sensitive detector system from Rigaku). The measurement time was reduced by factor of 100 with the application of a one-dimensional silicon strip detector (D/teX Ultra, Rigaku). One RSM measurement with the D/teX detector takes approximately 9 h with a speed equal to 2° min^−1^. RSMs around symmetrical and asymmetrical reflections were measured using a divergent height limiting slit, giving a beam height of 5 mm, and incident and receiving slits equal to 0.2 mm.

XRR measurements were carried out with an Ultima IV diffractometer (Rigaku) with Cu *K*α radiation. A high-resolution setup with a divergent height limiting slit equal to 2 mm, incident and receiving slits equal to 0.2 mm, and a scintillation counter detector was used for the measurements. Reflectivity curves were measured in the range 2θ = 0–6° with a step width of 0.001° and the rate during the measurement was equal to 0.02° min^−1^.

## 3. Theory

Reciprocal space maps contain a wealth of information on the structure of the thin film and the substrate. Some information can be extracted by means of simple operations with the RSM, like the peak position analysis; however, to obtain precise information about the structure is not so simple. By recording RSMs around both symmetric 004 and asymmetric 224 Bragg reflections (004 and 224 RSMs) for the same sample one can experimentally determine relaxation, lattice mismatch, lattice parameter, dislocation density and the tilt of the crystal planes of the layer. To estimate the degree of relaxation, two coupled RSMs were measured in grazing-incidence (sign −) and grazing-exit (sign +) geometries.

The peak positions can be used to determine the magnitude of the reciprocal lattice vector and hence in-plane and out-of-plane lattice period. With the help of Vegard’s law, the definition of relaxation and Hook’s law, the relaxation values can be found (Pietsch *et al.*, 2004 ▶).

We follow the approach developed by Kaganer *et al.* (1997 ▶), to perform the analysis of RSMs from partially relaxed structures, namely, to evaluate the dislocation density ρ and the correlation coefficient of dislocation, γ.

The shift of the layer peak relative to the substrate peak for 004 and 224 is
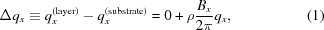


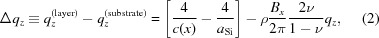
where 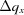 and 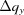 are the relative shifts of the layer peak relative to the substrate peak in coordinates in reciprocal space, 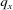 and 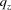 are the peak coordinates in reciprocal space, ρ is the dislocation density, *x* is the Ge concentration, and ν is the Poisson ratio. 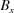 is a combination of the mismatch-releasing Burgers vector component and the Miller index of the considered reflection and is given by
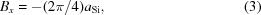
and the lattice constant of the unrelaxed layer is
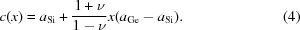
The first component on the right-hand side of equations (1) and (2) for 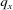 and 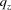 corresponds to the unrelaxed layer peak shift relative to the substrate peak; the second is related to the peak shift due to partial relaxation.

To estimate the theoretical dependence of the peak shape and width on the sample parameters, we make use of the model provided in the work of Kaganer *et al.* (1997 ▶). The shape of the layer peak is determined by the following equation:
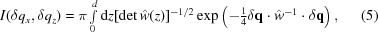
where 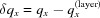 and 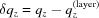 describe coordinates in reciprocal space relative to the peak position (maximum); 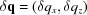; *d* is the layer thickness. Explicit expressions for the components 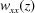, 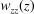, 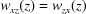 of the tensor 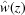 are provided by Kaganer *et al.* (1997 ▶).

The values that can be easily determined from the measured reciprocal space maps are the peak widths along axes 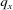 and 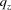, 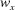 and 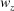, respectively. They are defined as the half-widths at half-height of the distributions 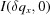 and 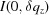.

Assuming 60° misfit dislocations and fixing the considered reflection (004 and 224 for the experimentally measured maps), one can represent the components 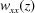, 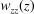, 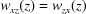 of the peak ellipse tensor 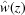 as functions of the parameters 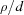 and 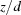:
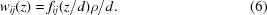
Then, integration over *z* can be carried out numerically in equation (5), and the widths are found to have the following values:
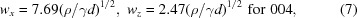


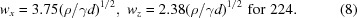
It is worth noting that, owing to the factorized form of equation (6), the numerical integration should be carried out just once for each type of reflection and the result will be valid for all values of the dislocation densities [that are described correctly by the model from Kaganer *et al.* (1997 ▶)].

Equations (7) and (8) provide the same dependence of 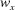 and 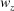 on the dislocation density ρ. Therefore, comparison of the measured and calculated values of these widths can provide information on the single parameter 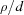 only (rather than on two independent parameters). However, good agreement of the measured and calculated values of the relation 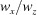 proves the validity of the 60° dislocation model for the considered samples.

The degree of relaxation can be found according to
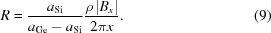
The lattice misfit can be calculated using the following equations:
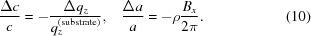
For calculations, the following values were used: *a*_Si_ = 5.43102 Å, *a*_Ge_ = 5.6579 Å and the equation *a*_SiGe_ = *a*_Si_(1−*x*) + *a*_Ge_ *x* − 0.026*x*(1−*x*) (De Salvador *et al.*, 2000 ▶).

## 4. Results and discussion

**Figure 1:**
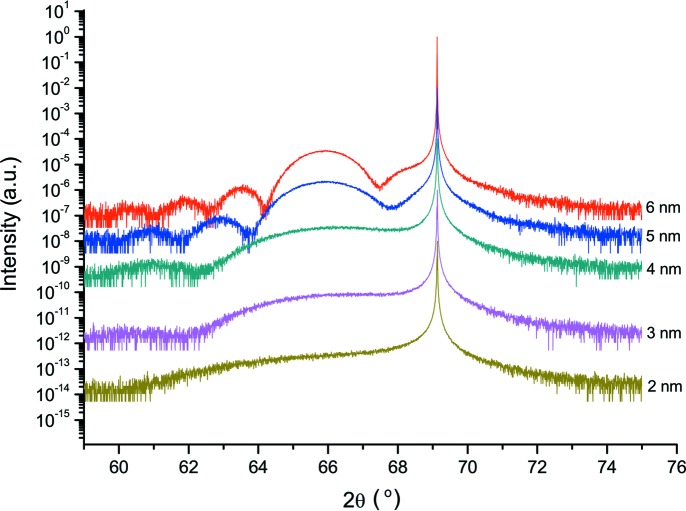
Measured rocking curves for the Si_0.4_Ge_0.6_ epilayers on Si substrates with thicknesses of 2–6 nm around the 004 Bragg reflection.

Fig. 1 ▶ demonstrates rocking curves from the super-thin samples with thicknesses of 2–6 nm. The measured high-resolution rocking curves demonstrate a good signal-to-noise ratio. It can be seen that the layer peak shoulder becomes longer when the thickness of the layer is decreased and the peak maximum becomes less visible. For the fully strained samples we can see the film thickness oscillations. Since the periodicity of thickness fringes corresponds to the thickness of the epilayer using these measurements, it is possible to evaluate the sample’s thickness with high accuracy. Using standard equations one may evaluate the thickness and concentration values (Pietsch *et al.*, 2004 ▶). The epilayer thicknesses obtained by HRXRD were checked using XRR measurements and TEM analysis. The observed oscillation on the XRR curves is caused by the SiGe and SiO_2_ layers. HRXRD is not sensitive to the SiO_2_. Results obtained from the HRXRD and XRR techniques and the TEM measurements are presented in Table 1 ▶ and they are in very good agreement within the error limits.

**Figure 2:**
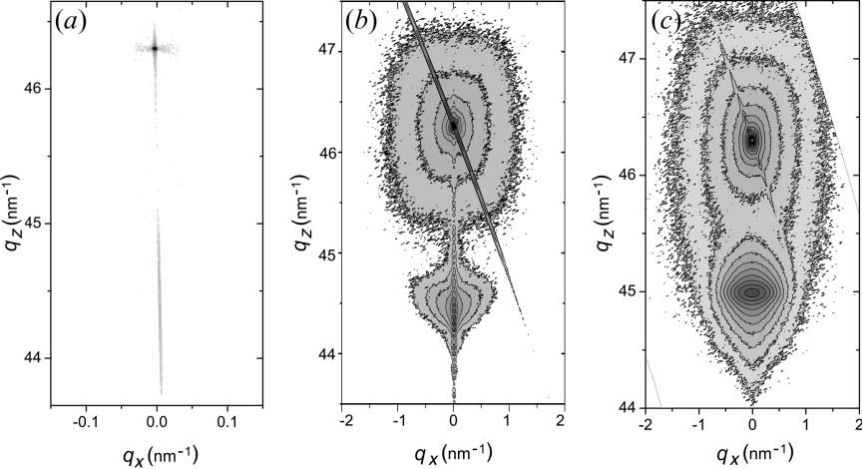
Measured RSMs of the symmetric 004 Bragg reflection for Si_0.4_Ge_0.6_/Si samples of thickness 6 nm (*a*), 29 nm (*b*) and 200 nm (*c*). The inclined stripes on the right-hand map are due to diffractometer optics and do not contain information about the investigated sample. 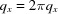. The intensity changes between isointensity contours by a factor of two.

In Fig. 2 ▶(*a*), the RSM of the symmetric 004 Bragg reflection for the sample with a thickness of approximately 6 nm is presented. The RSMs for the thinner samples look similar to Fig. 2 ▶(*a*). The peak width is narrow in the 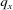 direction and elongated in the 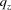 direction. According to Fig. 2 ▶, the sample with a thickness of about 6 nm has a single stripe along the 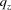 direction, which means no relaxation defects are present. The RSMs measured from the samples of thickness in the range 2–6 nm agree in shape with the dynamical diffraction theory predictions for pseudomorphic structures. This means that the diffraction is determined by the coherent scattering processes.

The RSMs of the symmetric 004 Bragg reflection in Figs. 2 ▶(*b*) and 2 ▶(*c*) for the samples with thicknesses of 29 and 200 nm exhibit a different behaviour, which is related to the beginning of the relaxation process. Fig. 2 ▶(*b*) demonstrates the presence of coherent and diffuse scattering simultaneously. This is an attribute of the first steps of the relaxation process. Further relaxation leads to the predominance of a diffuse scattering contribution, as is seen in Fig. 2 ▶(*c*). This kind of shape is predicted in Kaganer’s theory (Kaganer *et al.*, 1997 ▶), and supports the applicability of the approach. It is possible to see an elongated spot of diffracted intensity near the layer’s peak position which is related to the presence of diffuse scattering. It might be considered as a diffuse scattering onset, the centre of the elongated spot being related to the misfit dislocation density. The inclined stripes in Figs. 2 ▶(*b*) and 2 ▶(*c*) are due to diffractometer optics and do not alter information about the investigated sample.

**Figure 3:**
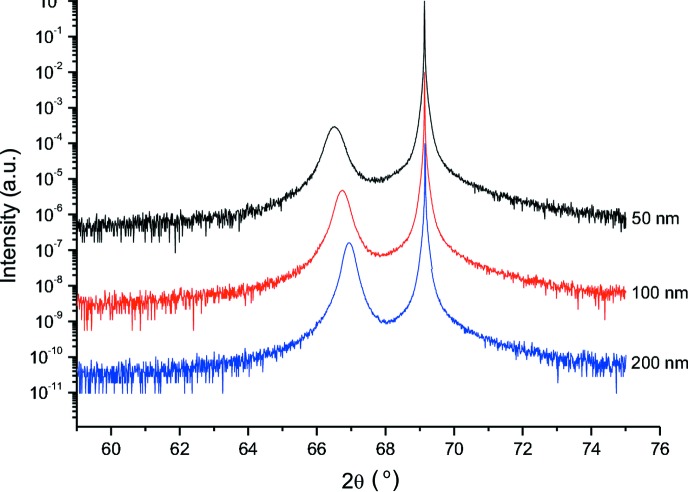
Measured rocking curves around the 004 Bragg reflection for Si_0.4_Ge_0.6_/Si samples of thickness 50, 100 and 200 nm.

In the case of the second set of Si_0.4_Ge_0.6_ samples, the epilayer thickness oscillations are not visible (see the rocking curves in Fig. 3 ▶).

**Figure 4:**
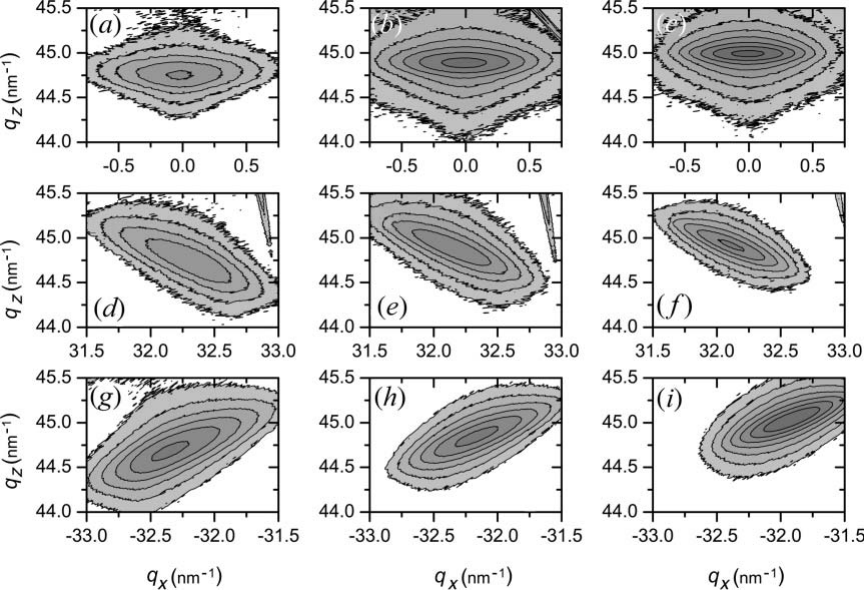
Contour plots of experimentally recorded intensity distribution from 004 (*a*)–(*c*), 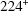 (*d*)–(*f*) and 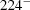 (*g*)–(*i*) reflections of Si_0.4_Ge_0.6_ epilayers on Si substrates. (*a*), (*d*) and (*g*) are for the sample with the layer thickness equal to 50 nm; (*b*), (*e*) and (*h*) for 100 nm; and (*c*), (*f*) and (*i*) for 200 nm. 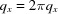. The intensity changes between isointensity contours by a factor of two.

The layer peaks of the RSMs measured for symmetrical and asymmetrical Bragg reflections (Fig. 4 ▶) are extended in the direction perpendicular to the corresponding diffraction vectors. Figs. 4 ▶(*a*)–4(*i*) present measured diffraction peaks of the 004 and 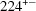 reflections for samples with thicknesses in the range 50–200 nm. According to Fig. 4 ▶, the peak widths in both directions (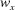 and 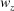) are decreasing with increasing layer thickness. This means that the relaxation degree increases with layer thickness. We may also see that the peak position on 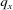 is changed; it moves to smaller 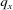 values. The X-ray measurements from samples of different thickness clearly demonstrate how the film changes state from pseudomorphic to partially relaxed. The relation between the film thickness and its relaxation is a result of different microscopic processes of dislocation propagation, nucleation and multiplication (Bolkhovityanov & Sokolov, 2012 ▶; Hu, 1991 ▶; Hull & Bean, 1992 ▶).

One may obtain the Ge concentration and relaxations from ω–2θ scans using the technique outlined by Zaumseil (1994 ▶). The layer thickness and dislocation density can be obtained from RSM peak shape using equations (1)–(8), if we assume that the dislocations are not correlated (γ = 1). For this evaluation reciprocal space maps around the 004 and 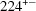 Bragg reflections were used. Using both peak shift values from the 224 maps one can determine ρ using equation (1), and then *x* from equation (2). The layer thickness *d* might be found according to equations (7) and (8) from measured peak widths. The results obtained are in good agreement with parameters obtained from reflectivity data and they are summarized in Table 2 ▶. The calculated values for Ge concentration are the same for the samples within the error of the calculation.

If we know the layer thickness and/or Ge concentration from other measurements (TEM, XRR), we may calculate dislocation density, relaxation and factor of correlation without making any assumptions about the dislocation nature (correlated or not) (see Table 2 ▶). In that case, the value 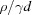 may be found according to equations (7) and  (8) from measured peak widths. Then from the peak shifts the value ρ according to equations (1) and (2) might be obtained. Next, the factor of dislocation correlation γ can be evaluated from the measured peak widths. We may see that the 60° misfit dislocations distributed in the films are uncorrelated (γ ≃ 1).

**Figure 5:**
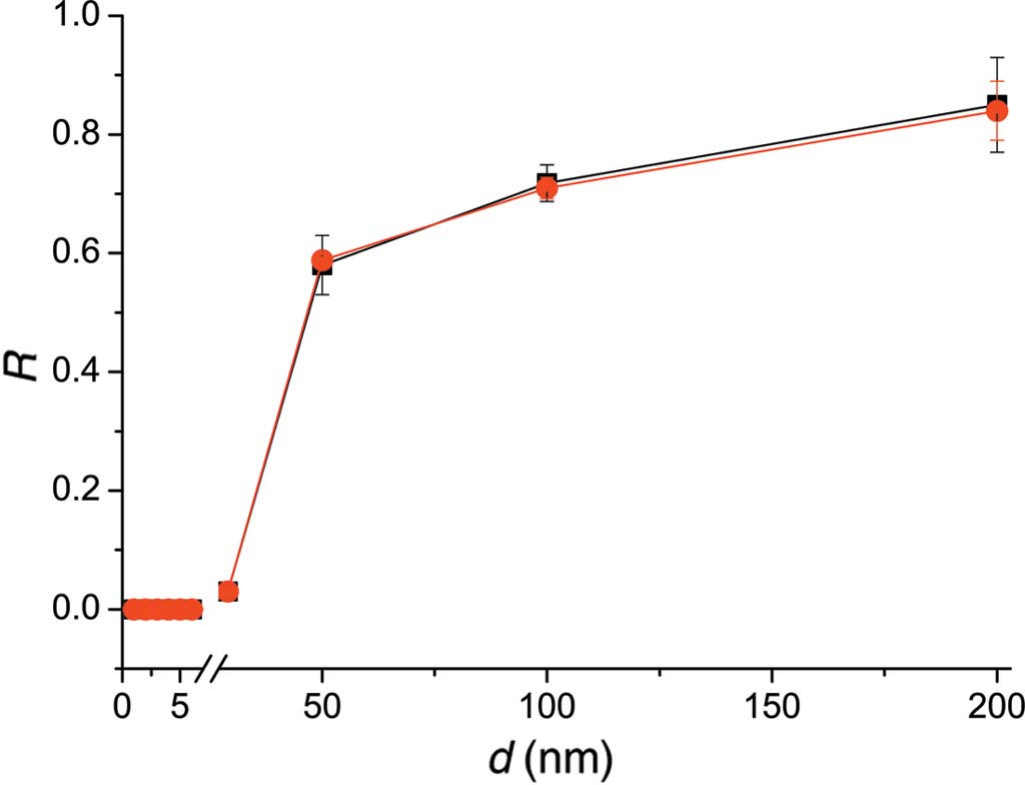
Relaxation *versus* layer thickness. The line with square marks is related to the relaxation values calculated from RSMs. The line with round marks corresponds to the relaxation values obtained using such parameters as thickness and Ge concentration as known.

For both cases considered above, on the basis of found parameters, the layer relaxation degree may be calculated using equation (9). Fig. 5 ▶ demonstrates the dependence of the relaxation on the thickness of the layer. We may see that the obtained relaxation values are more precise and the error bars are smaller in the case when thickness is considered as known during evaluation. This means that we may calculate the dislocation density more precisely.

The critical thickness according to the Matthews–Blakeslee model (Matthews & Blakeslee, 1974 ▶) for epitaxial Si_0.4_Ge_0.6_ films on Si(100) substrates in the case of 60° *a*/2 misfit dislocations is estimated to be *h*_cr_ ≃ 2.5 nm (Freund & Suresh, 2004 ▶). This is the thickness at which the first misfit dislocations should appear, *i.e.* the dislocation propagation becomes energetically profitable. However, it is known that the real dislocation propagation, which leads to the relaxation, becomes complicated owing to dislocation interaction, interaction with another defects and other processes. In such a case, the sample reconstruction takes place if the thickness is significantly large (Hull & Bean, 1992 ▶). The calculated critical thickness is much lower than the observed value since XRD is insensitive to propagation of individual dislocations, detecting only macroscopic changes in the sample, which became visible if large numbers of dislocations exist. In such a case, diffuse scattering is more important and leads to wide peaks on the RSMs. The contribution of coherent scattering decreases correspondingly. The example of an intermediate case is shown in Fig. 2 ▶(*b*). One may see the appearance of a diffuse spot and, at the same time, coherent scattering. This map is presented for the symmetric 004 Bragg reflection for the Si_0.4_Ge_0.6_/Si sample of thickness 29 nm. The relaxation degree is equal to 3%. This point is shown in Fig. 5 ▶. This thickness might be considered as a critical thickness for XRD.

## 5. Conclusions

The measured high-resolution rocking curves and reciprocal space maps from super-thin epitaxial layers demonstrate the good signal-to-noise ratio achievable with this laboratory-based diffractometer (Rigaku SmartLab). The data obtained on the basis of standard equations allowed the characterization of SiGe/Si structures (thickness range 2–6 nm). This means that the quality of measurements from modern laboratory instruments is now comparable to that which earlier was only available from synchrotron sources.

High-resolution X-ray reciprocal space mapping has been shown to be a reliable tool for comprehensive characterization of partially relaxed Si_0.4_Ge_0.6_ layers grown on a standard Si(001) substrate. Based on the theory developed by Kaganer *et al.* (1997 ▶), it is possible to find quantitatively, from high-resolution RSM peak shape (without thickness fringes), additional parameters such as misfit dislocation density and layer thickness. It is shown that both symmetric and asymmetric RSMs from such epitaxial layers contain enough information to obtain the Ge concentration, relaxation, layer thickness (without layer thickness oscillations), lattice misfit, dislocation density and factor of dislocation correlation based solely on the X-ray diffraction data obtained using a laboratory X-ray instrument. The thickness values obtained from XRD, XRR and TEM measurements are in very good agreement within the error limits.

## Figures and Tables

**Table 1:** The values of Ge concentration, *x*, and layer thickness, *d*, for super-thin samples evaluated from TEM, high-resolution rocking curve and reflectivity measurements

*d*_TEM_ (nm)	*x*	*d* (nm)	*d*_XRR_ (nm)	*x*_XRR_
2.0 (5)	0.60 (1)	1.40 (6)	1.99 (1)	0.601 (4)
3.0 (5)	0.596 (3)	2.62 (3)	2.99 (1)	0.599 (4)
4.0 (5)	0.603 (3)	2.96 (3)	4.25 (1)	0.599 (9)
5.0 (5)	0.600 (1)	5.12 (1)	4.91 (5)	0.599 (9)
6.0 (5)	0.602 (1)	6.50 (1)	6.31 (2)	0.601 (3)

**Table 2:** The values of Ge concentration, *x*, dislocation density, ρ, and layer thickness, *d*, for the second set of samples using high-resolution RSM data; the factor of dislocation correlation γ ≃ 1; the values of concentration *x*_XRR_ and thickness *d*_XRR_ using reflectivity measurements; and the thickness *d*_TEM_ obtained from TEM measurements

*d*_TEM_ (nm)	*x*	*d* (nm)	ρ (nm^−1^)	γ	*d*_XRR_ (nm)	*x*_XRR_
50.0 (5)	0.59 (5)	45 (15)	0.107 (8)	1.1800 (2)	49.61 (2)	0.60 (2)
100.0 (5)	0.61 (3)	100 (10)	0.135 (5)	1.0300 (3)	88.06 (9)	0.60 (3)
200.0 (5)	0.63 (6)	200 (60)	0.16 (1)	1.1100 (9)	199.0 (1)	0.60 (5)
